# Changes in Health Services Use Among Commercially Insured US Populations During the COVID-19 Pandemic

**DOI:** 10.1001/jamanetworkopen.2020.24984

**Published:** 2020-11-05

**Authors:** Christopher M. Whaley, Megan F. Pera, Jonathan Cantor, Jennie Chang, Julia Velasco, Heather K. Hagg, Neeraj Sood, Dena M. Bravata

**Affiliations:** 1 RAND Corporation, Santa Monica, California; 2 Castlight Health, San Francisco, California; 3 Sol Price School of Public Policy and Schaeffer Center, University of Southern California, Los Angeles; 4 National Bureau for Economic Research, Cambridge, Massachusetts; 5 Center for Primary Care and Outcomes Research, Stanford, California

## Abstract

**Importance:**

The coronavirus disease 2019 (COVID-19) pandemic has placed unprecedented strain on patients and health care professionals and institutions, but the association of the pandemic with use of preventive, elective, and nonelective care, as well as potential disparities in use of health care, remain unknown.

**Objective:**

To examine changes in health care use during the first 2 months of the COVID-19 pandemic in March and April of 2020 relative to March and April of 2019 and 2018, and to examine whether changes in use differ by patient’s zip code–level race/ethnicity or income.

**Design, Setting, and Participants:**

This cross-sectional study analyzed health insurance claims for patients from all 50 US states who receive health insurance through their employers. Changes in use of preventive services, nonelective care, elective procedures, prescription drugs, in-person office visits, and telemedicine visits were examined during the first 2 months of the COVID-19 pandemic in 2020 relative to existing trends in 2019 and 2018. Disparities in the association of the pandemic with health care use based on patient’s zip code–level race and income were also examined.

**Results:**

Data from 5.6, 6.4, and 6.8 million US individuals with employer-sponsored insurance in 2018, 2019, and 2020, respectively, were analyzed. Patient demographics were similar in all 3 years (mean [SD] age, 34.3 [18.6] years in 2018, 34.3 [18.5] years in 2019, and 34.5 [18.5] years in 2020); 50.0% women in 2018, 49.5% women in 2019, and 49.5% women in 2020). In March and April 2020, regression-adjusted use rate per 10 000 persons changed by −28.2 (95% CI, −30.5 to −25.9) and −64.5 (95% CI, −66.8 to −62.2) for colonoscopies; −149.1 (95% CI, −162.0 to −16.2) and −342.1 (95% CI, −355.0 to −329.2) for mammograms; −60.0 (95% CI, −63.3 to −54.7) and −118.1 (95% CI, −112.4 to −113.9) for hemoglobin A_1c_ tests; −300.5 (95% CI, −346.5 to −254.5) and −369.0 (95% CI, −414.7 to −323.4) for child vaccines; −4.6 (95% CI, −5.3 to −3.9) and −10.9 (95% CI, −11.6 to −10.2) for musculoskeletal surgery; −1.1 (95% CI, −1.4 to −0.7) and −3.4 (95% CI, −3.8 to −3.0) for cataract surgery; −13.4 (95% CI, −14.6 to −12.2) and −31.4 (95% CI, −32.6 to −30.2) for magnetic resonance imaging; and −581.1 (95% CI, −612.9 to −549.3) and −1465 (95% CI, −1496 to −1433) for in-person office visits. Use of telemedicine services increased by 227.9 (95% CI, 221.7 to 234.1) per 10 000 persons and 641.6 (95% CI, 635.5 to 647.8) per 10 000 persons. Patients living in zip codes with lower-income or majority racial/ethnic minority populations experienced smaller reductions in in-person visits (≥80% racial/ethnic minority zip code: 200.0 per 10 000 [95% CI, 128.9-270.1]; 79%-21% racial/ethnic minority zip code: 54.2 per 10 000 [95% CI, 33.6-74.9]) but also had lower rates of adoption of telemedicine (≥80% racial/ethnic minority zip code: −71.6 per 10 000 [95% CI, −87.6 to −55.5]; 79%-21% racial/ethnic minority zip code: −15.1 per 10 000 [95% CI, −19.8 to −10.4]).

**Conclusions and Relevance:**

In this cross-sectional study of a large US population with employer-sponsored insurance, the first 2 months of the COVID-19 pandemic were associated with dramatic reductions in the use of preventive and elective care. Use of telemedicine increased rapidly but not enough to account for reductions in in-person primary care visits. Race and income disparities at the zip code level exist in use of telemedicine.

## Key Points

**Question:**

How did health services use among commercially insured populations change during the initial phase of the coronavirus disease 2019 (COVID-19) pandemic in the United States?

**Findings:**

This cross-sectional study with a claims-based analysis of 6.8 million commercially insured individuals found that during the initial phase of the COVID-19 epidemic in March and April of 2020, patients significantly reduced use of preventive and elective care and increased use of telemedicine but not enough to offset reductions in in-person care. Racial/ethnic and income disparities were seen in changes in use of in-person care and telemedicine.

**Meaning:**

In this study, the initial 2 months of the COVID-19 pandemic were associated with large reductions in use of health services; future policy initiatives should ensure that these reductions do not adversely affect patient health.

## Teaser

This cross-sectional study analyzes health insurance claims among commercially-insured individuals to asess changes in the use of health services in the Unted States during the initial phase of the COVID-19 pandemic.

## Introduction

The coronavirus disease 2019 (COVID-19) pandemic has placed tremendous pressure on patients and health care professionals and institutions, both in the United States and worldwide.^1,2,3,4,5^ US health care practitioners have reported significant decreases in non-COVID–related health care use since the beginning of the pandemic.^6,7^ Recent estimates from the US Centers for Disease Control and Prevention and others suggest a decline in emergency department visits and outpatient visits associated with the pandemic.^6,8,9^ The reduction in visits is likely due to patient fears of infection and reduced access to traditional in-person health care owing to public health regulations that have restricted elective procedures.^7,10^ However, empirical estimates of the size of the decline in utilization are limited.^11,12,13^ Existing studies have typically used data from a single health system or geographic market to observe changes in utilization and have not consistently documented changes in specific types of care that have been affected by the COVID-19 pandemic.^6,7^ They have also documented the rapid increase in telemedicine visits, defined as the remote diagnosis and treatment of patients by means of telecommunications technologies.^14^ The lack of national estimates of telemedicine use is a critical gap in the literature given that the US Centers for Disease Control and Prevention recommends the use of virtual visits to counteract concerns of transmission of COVID-19.^8^

In this study, we examined whether the first 2 months of the COVID-19 pandemic were associated with changes in non-COVID health care use among a large population of individuals with employer-sponsored insurance. Specifically, we sought to characterize the use of preventive services (eg, pediatric vaccinations), elective services (eg, orthopedic surgery), and nonelective services (eg, labor and delivery care) in March and April 2020 compared with March and April 2018 and March and April 2019. We also examined changes in the number of telemedicine visits. Given concerns that the COVID-19 epidemic may affect at-risk populations disproportionately,^15,16,17^ we quantified the changes in use by the patient’s zip code’s race and income.

## Methods

To assess national changes in health care use during the initial phase of the COVID-19 pandemic, this cross-sectional study used health insurance claims data from 2018 to 2020 collected by Castlight Health. Castlight Health aggregates medical and pharmaceutical claims from self-insured employers and health plans that purchase access to their transparency tools and benefits platform. Approximately 200 employers with employees in all US states contributed data. These employers represented several industries and varied in the number of employees, from 500 to more than 500 000. The data included claims from 5.6 million individuals in 2018, 6.4 million individuals in 2019, and 6.8 million individuals in 2020. The study population accounted for $50.1 billion in health care spending from January 1, 2018, to April 30, 2020. The eAppendix in the Supplement provides further detail on the geographic areas and industries represented in the study. This study and a waiver of informed consent was approved by the RAND institutional review board, and a preregistration plan was filed on the Open Sciences Framework.^18^ Informed consent was waived because the data were collected for nonresearch purposes and are deidentified. This study followed the Strengthening the Reporting of Observational Studies in Epidemiology (STROBE) reporting guideline.

These claims data were used to examine changes in the use of health care services and specific procedures. Monthly use of health care services was measured by calculating the number of persons who received each procedure per 10 000 eligible persons in each month. Procedures were defined using the IBM Watson Health procedure categories.^19,20^ Incidence rates were calculated for all grouped procedures and 10 medical procedures of specific interest. The 10 procedures included preventive services (eg, mammograms per woman aged 46-64 years, colonoscopies per person aged 45-64 years, vaccinations per child younger than 2 years, and hemoglobin A_1c_ [HbA_1c_] tests), nonelective care (eg, labor and delivery services per woman aged 19-45 years, chemotherapy, and percutaneous transluminal coronary angioplasty), and elective procedures (magnetic resonance imaging [MRI], cataract surgeries, and musculoskeletal surgeries).

Use of prescription drugs for 3 drug classes that are prescribed to manage chronic conditions—antidiabetes medications, asthma medications, and statins—was also examined. Drug class groupings were obtained from First Databank.^21^ Finally, trends in the use of office visits and telemedicine services were examined. Patient use of telemedicine was defined using procedure codes and site of service fields (eAppendix in the Supplement). The primary diagnosis code for the patient’s visit was also identified.

### Statistical Analysis

To evaluate trends in health care use, the study aggregated the outcomes at the monthly level from 2018 to 2020 and calculated monthly utilization per 10 000 enrollees. The study compared each outcome in March and April 2020 to the same outcome in the previous 2 years. March 2020 was selected as the start of the study period because the US declared the COVID-19 outbreak a national emergency in this month.^22^ Thus, the study measured the changes in health care use at the initial phase of the COVID-19 pandemic in the US. The study also compared the outcome measures from January and February of 2020 with those in January and February of 2019 or 2018. These changes reflect secular trends only as they occurred prior to the start of the COVID-19 pandemic. Therefore, the difference between the change in use between March and April 2020 and March and April in 2019 and 2018 and the change in use between January and February 2020 and January and February in 2019 and 2018 isolate the association of the initial phase of the COVID-19 pandemic with health care use.

This study also estimated multivariable regressions that test for the change in each outcome at the patient age, gender, state, year, and month level that occurred in March and April 2020. These regressions, which are further described in the eAppendix in the Supplement, include fixed effects for calendar year and month and include controls for patient age, patient sex, and patient state. To assess these associations from the perspective of the typical patient, regressions were weighted by the number of eligible members in each cell. To further assess whether trends were due to changes in patient composition, we examined trends in patient sex, age, geographic region, and risk score, which were measured using the Verisk DxCG risk score model.^23^ The regression models used robust standard errors. All significance testing was 2-sided with a significance threshold of *P* < .05.

The study also examined whether changes in preventive care, in-person office visits, and telemedicine varied by the income and race of the patient’s 5-digit zip code because of disparities in mobility responses to COVID-19,^24,25^ use of telemedicine,^26^ and health outcomes.^27^ The study lacked patient-specific income and race/ethnicity data and thus obtained zip code–level measures from the American Community Survey.^28^ The study classified zip codes by income relative to the federal poverty line (FPL) (0%-200%, 200%-400%, and >400% of FPL). The study categorized zip codes by the share of residents who belong to racial/ethnic minority groups (≥80% minority population, 79%-21% minority population, and ≥80% minority population).^29^ For this analysis, the March and April 2020 periods were pooled into a single measure for ease of interpretation.

To ensure the robustness of results, the study conducted a number of sensitivity tests. First, whereas the main analysis examined changes in health care use, a sensitivity analysis was used that examined changes in monthly spending. Second, whereas our main results highlight changes in use rates for select procedures of interest, a sensitivity analysis was used that examined growth trends for all procedures combined. Stata, version 16 (StataCorp LLC) was used for all statistical analyses.

## Results

The study population had similar age (mean [SD], 34.3 [18.6] years in 2018, 34.3 [18.5] years in 2019, and 34.5 [18.5] years in 2020), sex (50.0% women in 2018, 49.5% women in 2019, and 49.5% women in 2020), patient risk score (mean [SD] score, 1.27 [3.71] in 2018, 1.21 [3.69] in 2019, and 1.19 [3.29] in 2020), and geographic distribution across each of the 3 study years (South: 40.6% in 2018, 40.0% in 2019, and 39.6% in 2020; Midwest: 23.0% in 2018, 23.2% in 2019, and 24.4% in 2020; Northeast: 10.4% in 2018, 10.8% in 2019, and 11.4% in 2020; West: 26.0% in 2018, 25.9% in 2019, and 24.7% in 2020) (Table 1). Per-person medical spending was also similar in each year (mean [SD], $300 [$3707] in 2018, $316 [$3983] in 2019, and $320 [$3822] in 2020).

**Table 1.** Characteristics of Study Population

Characteristic	Enrolled persons, No. (%)		
	2018 (n = 5 608 888)	2019 (n = 6 389 425)	2020 (n = 6 953 508)^a^
Patient demographics			
Women	2 802 962 (50.0)	3 160 508 (49.5)	3 437 885 (49.5)
Mean (SD) age, y	34.3 (18.6)	34.3 (18.5)	34.5 (18.5)
Census region			
South	2 278 894 (40.6)	2 558 103 (40.0)	2 753 640 (39.6)
Midwest	1 288 049 (23.0)	1 485 304 (23.2)	1 698 293 (24.4)
Northeast	585 061 (10.4)	688 062 (10.8)	791 257 (11.4)
West	1 456 794 (26.0)	1 657 956 (25.9)	1 710 318 (24.7)
Risk score, mean (SD)^b^	1.27 (3.71)	1.21 (3.69)	1.19 (3.29)
Percentage of persons in zip code with <80% White residents, mean (SD)	52.3 (18.7)	51.6 (19.3)	51.5 (18.9)
Income (census-tract level), FPL^c^			
0-200%	1 430 266 (25.5)	1 661 251 (26.0)	1 745 331 (25.1)
201%-400%	3 483 119 (62.1)	3 961 444 (62.0)	4 199 919 (60.4)
>400%	903 031 (16.1)	1 022 308 (16.0)	1 070 840 (15.4)
Population use and spending characteristics			
Cost per claim, January-February, mean (SD), $	168 (427)	173 (559)	176 (354)
Member claims per month, January-February, mean (SD)	1.78 (0.17)	1.83 (0.10)	1.82 (0.11)
Per-member, per-month medical spending, January-February, mean (SD), $	300 (3707)	316 (3983)	320 (3822)

Abbreviation: FPL, federal poverty line.

^a^ January to March only.

^b^ Risk score is calculated using the DxCG risk score.

^c^ FPL indicates 2018 federal poverty line, which is $26 200 for a household of 4.

**Figure 1.**
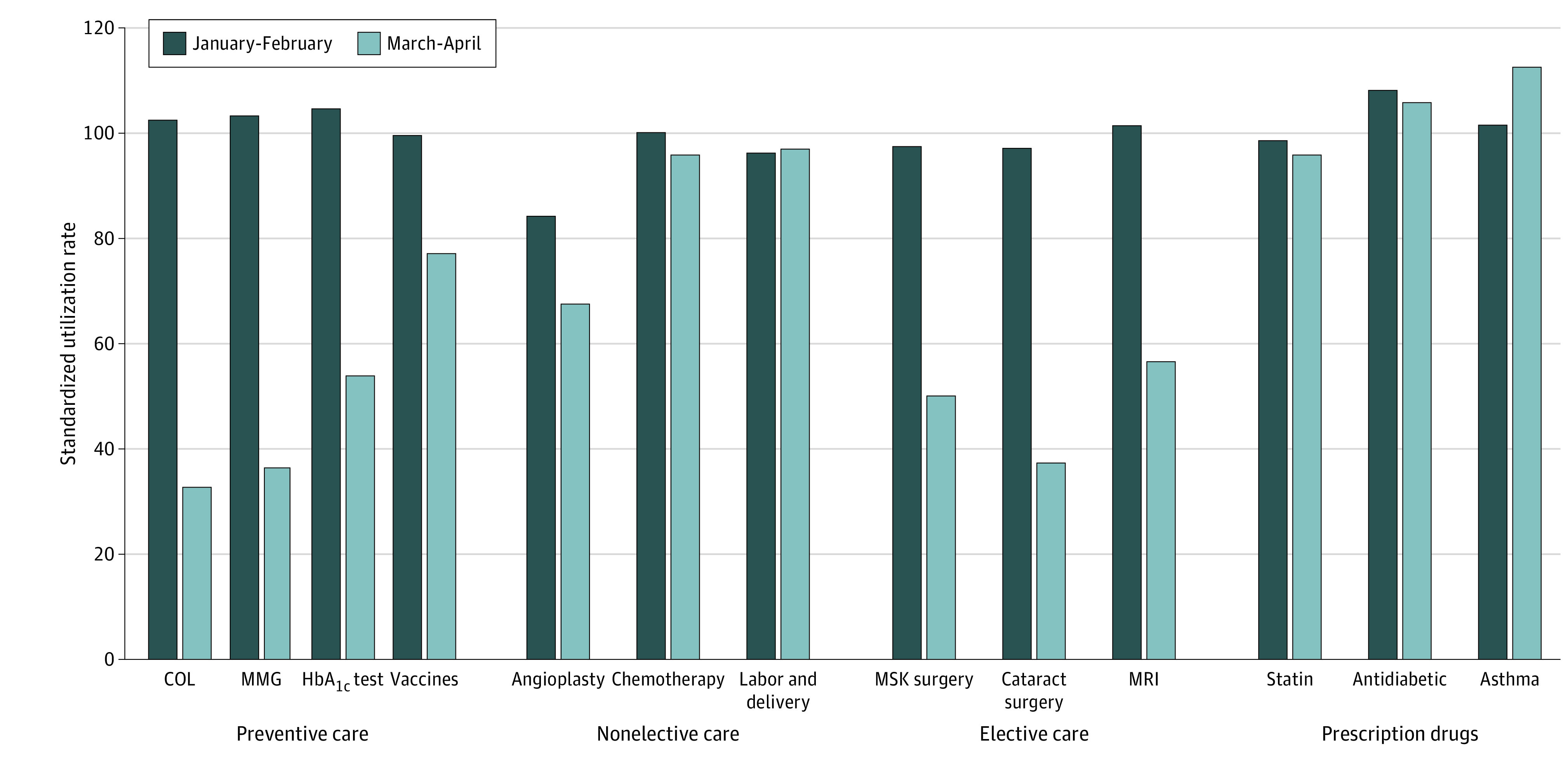
Unadjusted Utilization of Preventive, Nonelective, Elective, and Pharmaceutical Services in January/February and March/April 2020 Compared With 2019 The colonoscopy (COL) population was limited to ages 46 to 64 years; mammogram (MMG) population, to women aged 46 to 64 years; vaccine population, to children aged 0 to 2 years; and labor and delivery population, to women aged 19 to 45 years. HbA_1c_ indicates hemoglobin A_1c_; MMG, mammogram; MRI, magnetic resonance imaging; and MSK, musculoskeletal.

Figure 1 presents unadjusted trends in preventive care, nonelective medical services, elective medical services, and prescription drugs in January and February or March and April and for both 2019 and 2020. Comparisons between March and April 2018 and 2020 are presented in the eFigure in the Supplement. Use rates observed in 2019 are normalized to 100 so that changes between 2019 and 2020 can be interpreted as percentage changes. Among persons aged 46 to 64 years, use of colonoscopy increased by 2.3% in January and February 2020 relative to rates observed in 2019. For March and April 2020, use of colonoscopy decreased by 69.6% relative to rates observed in March and April 2019. The relative difference between the January and February and March and April differences was a 71.9% reduction in the use of colonoscopies. Relative reductions of 67.0% for mammograms among women aged 46 to 64 years, 50.7% for HbA_1c_ tests, 22.3% for vaccines among children aged 0 to 2 years, and 16.8% for angioplasty were observed in March and April 2020. No meaningful changes in labor and delivery rates (0.7% increase) were observed; a 4.1% relative decrease in chemotherapy treatments was observed. There were relative use decreases of 47.4% for musculoskeletal surgery, 59.8% for cataract surgery, and 45.0% for MRIs. Unadjusted prescription drug use decreased by 2.8% for statins and 2.3% for antidiabetic medications, and use increased by 11.1% for asthma medications.

For primary care services in March 2020, after regression adjustment, use of colonoscopy services had a change of −28.2 persons per 10 000 persons aged 46 to 64 years (95% CI, −30.5 to −25.9) compared with other periods (Table 2). Based on the March 2019 utilization rate of 64.3 persons per 10 000 eligible persons, the reduction in utilization translates to a 43.9% reduction in the use of colonoscopies in March 2020. This difference increased to a 64.5 per-10 000-person difference in April 2020 (95% CI, −66.8 to −62.2), a relative reduction of 92.9%. Similar relative reductions were observed for other services: 41.6% and 90.4% for mammograms among women ages 46 to 64 in March and April 2020, respectively (absolute reduction: −149.1 per 10 000; 95% CI, −162.0 to −16.2 in March and −342.1 per 10 000; 95% CI, −355.0 to −329.2 in April), 35.1% and 68.9% for HbA1c tests (absolute reductions: −60.0 per 10 000; 95% CI, −63.3 to −54.7 in March and −118.1 per 10 000; 95% CI, −112.4 to −113.9 in April), and 18.0% and 22.6% for vaccines among children aged 0 to 2 years (absolute reductions: −300.5 per 10 000; 95% CI, −346.5 to −254.5 and −369.0 per 10 000; 95% CI, −414.7 to −323.4).

**Table 2.** Multivariable Regression Results: Change in Health Care Service Use in March 2020^a^

Variable	Change per 10 000 persons (95% CI)												
	Preventive care				Nonelective care			Elective care			Prescription drug		
	Colonoscopy^b^	Mammogram^c^	HbA_1C_ test	Vaccines^d^	Angioplasty	Chemotherapy	Labor and delivery^e^	MSK surgery	Cataract surgery	MRI	Statin	Antidiabetic	Asthma
March 2020	−28.21 (−30.51 to −25.91)^f^	−149.1 (−162.0 to −136.2)^f^	−58.97 (−63.26 to −54.69)^f^	−300.5 (−346.5 to −254.5)^f^	−0.131 (−0.268 to 0.00638)	−0.685 (−1.368 to −0.00180)^g^	−0.161 (−2.320 to 1.997)	−4.629 (−5.336 to −3.922)^f^	−1.063 (−1.432 to −0.695)^f^	−13.39 (−14.58 to −12.21)^f^	6.450 (−8.580 to 21.48)	2.060 (−1.337 to 5.457)	57.14 (52.09 to 62.18)^f^
April 2020	−64.45 (−66.75 to −62.16)^f^	−342.1 (−355.0 to −329.2)^f^	−118.1 (−122.4 to −113.9)^f^	−369.0 (−414.7 to −323.4)^f^	−0.278 (−0.415 to −0.141)^g^	−1.445 (−2.126 to −0.764)^g^	−1.219 (−3.369 to 0.931)	−10.86 (−11.57 to −10.16)^f^	−3.401 (−3.768 to −3.034)^f^	−31.39 (−32.57 to −30.20)^f^	−20.72 (−35.70 to −5.742)^f^	−3.603 (−6.989 to −0.218)^g^	−4.542 (−9.570 to 0.486)
Observations, No.	5712	2856	19 989	2853	19 989	19 989	2856	19 989	19 989	19 989	19 989	19 989	19 989
*R*^2^	0.723	0.814	0.914	0.909	0.472	0.865	0.558	0.795	0.807	0.879	0.841	0.800	0.727
March 2019 use rate	64.3	358.4	168.0	1665.6	0.9	18.9	34.4	16.0	3.4	47.8	248.7	52.7	174.8
April 2019 use rate	69.4	378.5	171.3	1632.1	0.8	19.6	34.9	16.4	3.7	50.1	257.1	54.7	177.3
Relative change, %													
Between March 2019 and March 2020	−43.9	−41.6	−35.1	−18.0	−15.2	−3.6	−0.5	−28.9	−31.3	−28.0	2.6	3.9	32.7
Between April 2019 and April 2020	−92.9	−90.4	−68.9	−22.6	−33.0	−7.4	−3.5	−66.0	−91.1	−62.6	−8.1	−6.6	−2.6

Abbreviation: MSK, musculoskeletal.

^a^ This table shows regression-adjusted differences in use rates of preventive, nonelective, elective, and pharmaceutical care in March 2020 and April 2020, relative to the 2018 to 2020 time period. The dependent variable in each column is the monthly number of persons per 10 000 eligible persons with the respective procedure. Regression models include fixed-effect controls for year and month, state, patient sex, and age category (categorized as 0-2, 3-18, 19-26, 27-45, and 46-64 years).

^b^ Limited to ages 46 to 64 years.

^c^ Limited to women aged 46 to 64 years.

^d^ Limited to children aged 0 to 2 years.

^e^ Limited to women aged 19 to 45 years.

^f^ *P* < .05.

^g^ *P* < .01.

For nonelective services, use of angioplasty services decreased by 0.1 persons per 10 000 persons (95% CI, −0.3 to −0.1) in March 2020, and by 0.3 persons per 10 000 (95% CI, −0.42 to −0.14) in April 2020, for relative reductions of 15.2% and 33.0% when compared with the March and April 2019 rates of 0.9 and 0.8 procedures per 10 000 persons, respectively. Chemotherapy declined by a relative 3.6% (absolute reduction: −0.7 per 10 000; 95% CI, −1.4 to −0.0) in March 2020 and 7.4% (absolute reduction: −1.4 per 10 000. 95% CI, −2.1 to −0.8) in April 2020. The study did not find statistically significant decreases in labor and delivery among women aged 19 to 45 years.

For elective services, use of musculoskeletal surgery decreased by a relative 28.9% in March 2020 and 66.0% in April 2020 (absolute reductions: −4.6 per 10 000; 95% CI, −5.3 to −3.9 in March and −10.9 per 10 000; 95% CI, −11.6 to −10.2 in April), 31.3 and 9.91% for cataract surgery (absolute reductions: −1.1 per 10 000; 95% CI, −1.4 to −0.7 in March and −3.4 per 10 000; 95% CI, −3.8 to −3.0 in April), and 28.0% and 62.6% for MRIs (absolute reductions: −13.4 per 10 000 in March; 95% CI, −14.6 to −12.2 and −31.4 per 10 000 in April; 95% CI, −32.6 to −30.2). This study did not find statistically significant changes in the use of statins or antidiabetic medications in March 2020 but did find respective decreases of 8.1% and 6.6% in April 2020 (absolute differences −20.7 per 10 000; 95% CI, −35.7 to −5.7 in March and −3.6 per 10 000; 95% CI, −7.0 to −0.2 in April). Use of asthma medications increased by a relative 32.7% in March 2020 (absolute increase: 57.1 per 10 000; 95% CI, 52.1 to 62.1). Across all 145 procedures, 14.0 fewer persons per 10 000 sought care in March 2020 (95% CI, −14.8 to −13.2), which translates to a 22.7% decrease in utilization, and 32.2 fewer persons per 10 000 sought care in April 2020 (95% CI, −33.0 to −31.4), a 51.5% relative reduction (eTable 3 in the Supplement). Relative to existing time trends, medical spending decreased by 21.5% in March 2020 and by 45.5% in April 2020 (eTable 4 in the Supplement).

**Figure 2.**
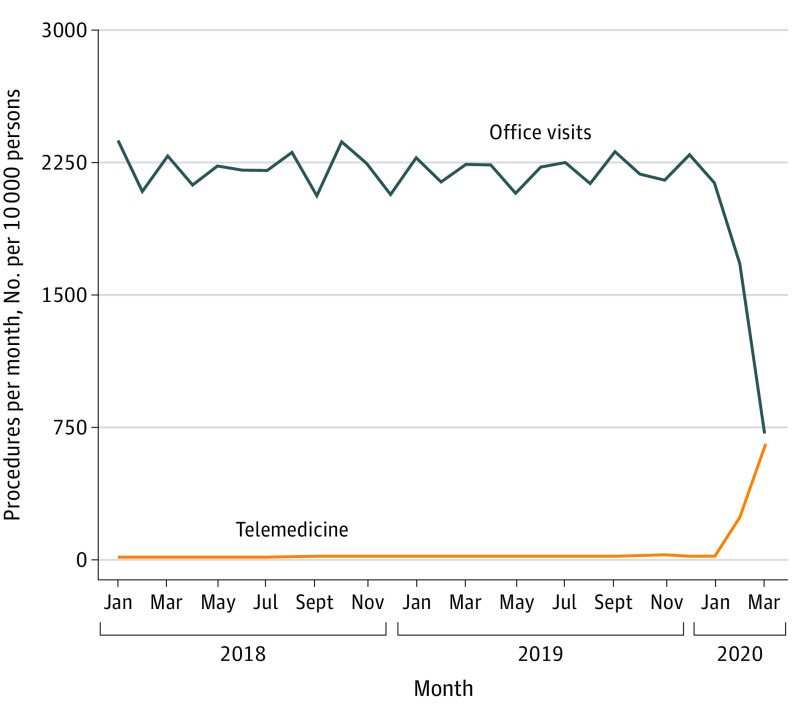
Trends in Use of Office Visits and Telemedicine This figure presents trends in the monthly number of patients with an office visit (blue line) or a telemedicine visit (orange line) per 10 000 persons.

A dramatic increase in the use of telemedicine services was observed in March and April 2020 compared with all prior time periods (Figure 2). Relative to March 2019, the number of telemedicine visits per 10 000 persons increased by 221.1 visits per 10 000 persons, from 17.4 in 2019 to 239.1 in March 2020, a relative increase of 1270%. Relative to April 2019, telemedicine visits increased by 635.5 visits per 10 000 persons in April 2020, a relative increase of 4081%. In-person office visits decreased by smaller relative amounts (25.0% in March 2020 and 68.0% in April 2020) but larger absolute amounts (560.1 visits per 10 000 persons in March 2020 and 1520.8 visits per 10 000 persons in April 2020). Thus, the March and April 2020 increase in the use of telemedicine services offset only 40% and 42%, respectively, the reduction in office visits. In April 2020, 48% of combined in-person and telemedicine visit were delivered virtually. Similar results were obtained when using a regression approach (eTable 5 in the Supplement). Per-10 000-person regression-adjusted use of in-person office visits decreased by 581.1 (95% CI, −612.9 to −549.3) in March 2020 and by 1465 (95% CI, −1496 to −1433) in April 2020. Use of telemedicine services increased by 227.9 (95% CI, 221.7 to 234.1) and 641.6 (95% CI, 635.5 to 647.8) visits in March and April 2020, respectively.

**Figure 3.**
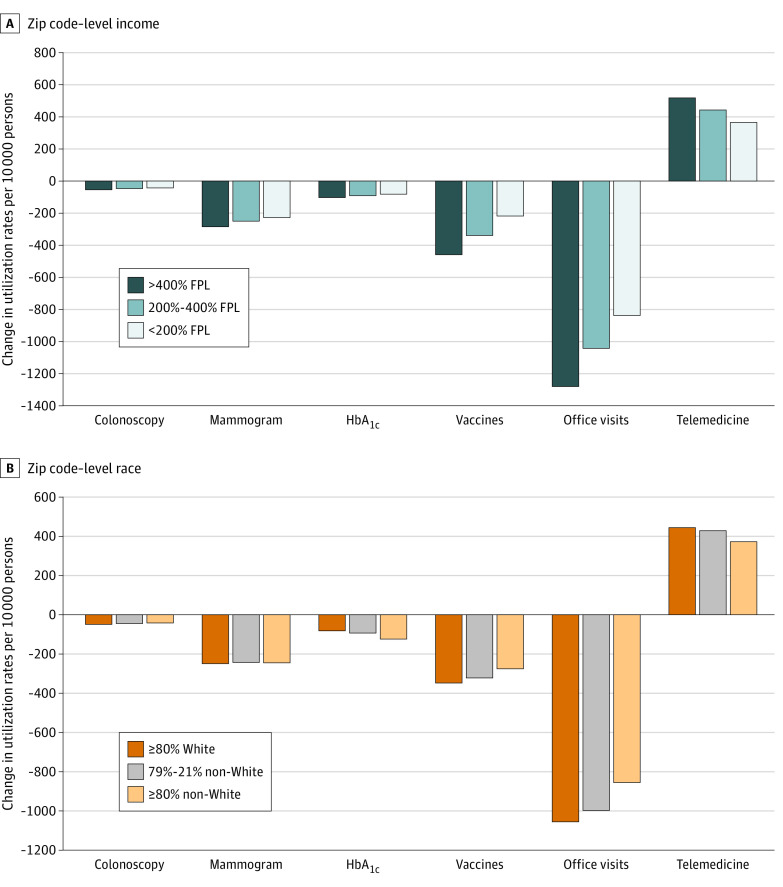
Differences in Change in Preventive Care, Office-Based Visits, and Telehealth by Patient Zip Code–Level Income and Race This figure shows regression-adjusted changes in the monthly per-10 000 eligible persons use of preventive care (colonoscopy, mammograms, hemoglobin A_1c_ [HbA_1c_] tests, vaccines), office visits, and telemedicine in March 2020. Panel A presents results by zip code income relative to the federal poverty line (FPL), and panel B presents results based on the share of residents that are White individuals or members of minority ethnic/racial groups (non-White). Regression models include fixed-effect controls for year and month, state, patient sex, and age category (categorized as 0-2, 3-18, 19-26, 27-45, and 46-64 years). The colonoscopy population is limited to ages 46 to 64 years; mammogram population, to women aged 46 to 64 years; and vaccine population, to children aged 0 to 2 years.

Figure 3 presents regression-adjusted changes in the use of preventive care, office visits, and telemedicine visits by zip code–level income (Figure 3A) and percent of population who belong to racial/ethnic minority groups (Figure 3B). In March and April 2020, patients residing in zip codes with a mean income greater than 400% of FPL reduced use of colonoscopies by 54.8 persons per 10 000 persons among individuals aged 46 to 64 years, compared with a 46.9-person reduction in zip codes between 200% and 400% of FPL and 40.7-person reduction for zip codes below 200% of FPL. Similar differences in trends were observed for mammograms, HbA_1c_ tests, and vaccines, with patients in lower-income zip codes reducing preventive care less than patients in higher-income zip codes in March and April 2020. However, these differences were not large enough to offset pre-COVID disparities in the use of preventive care (full regression results in eTable 6 in the Supplement). For office visits, smaller reductions in care were observed for patients in lower-income zip codes, but so were lower rates of telemedicine increases in March and April 2020. Compared with those in zip codes with 80% or more White residents, patients in zip codes with 80% or more residents who belong to racial/ethnic minority groups had smaller reductions in the use of in-person office visits (absolute difference: 200.0 per 10 000; 95% CI, 128.9 to 270.1) but also smaller increases in the use of telemedicine (absolute difference: −71.6 per 10 000; 95% CI, −87.6 to −55.5). For those in zip codes with 79% to 21% residents who belong to racial/ethnic minority groups, relative use of in-person office visits increased by 54.2 per 10 000 (95% CI, 33.6 to 74.9), and relative use of telemedicine decreased by 15.1 per 10 000 (95% CI, −19.8 to −10.4).

## Discussion

The COVID-19 pandemic and government responses to the pandemic has disrupted daily life in the United States and worldwide. The health care delivery system has been uniquely affected. Hospitals and emergency departments have faced unprecedented stresses treating COVID-19 patients, often with shortages of essential equipment—including personal protective equipment, intensive care unit beds, and ventilators. At the same time, many health care practitioners and patients have cancelled or deferred care.

We used a nationwide sample of patients with private insurance to examine the association of the initial phase of the COVID-19 pandemic with health care use. We found dramatic decreases in the use of health services; overall health care use declined by 23% in March 2020 and by 52% in April 2020 relative to existing time trends. We observed large reductions in use of high-value preventive care and many elective procedures. We found small or no changes in the use of nonelective care and no change or an increase in use of prescription drugs.

A key area where we observed large increases (more than 1000% in March 2020 and more than 4000% in April 2020) was in the use of telemedicine services. In April 2020, 48% of consultation visits were delivered virtually. However, the increase in telemedicine use offset only approximately 40% of the declines in in-person office visits, suggesting that many primary care needs may be going unmet. The increased use in telemedicine could be due to changes in financing, licensing, and adaptions by patients, insurance companies, and health professionals.^30,31,32^ It is important to note that many of these policies were only instituted in March, and thus there is still an opportunity for the unmet need gap to be further filled by telemedicine as the pandemic continues. Others have reported that the COVID-19 pandemic and its associated economic consequences are putting enormous stresses on the mental well-being of the US population.^33^ Moreover, behavioral health care, which does not rely on physical examination, is uniquely suited to delivery via telemedicine channels.^34^

Finally, we found smaller reductions in care use and lower rates of telemedicine use among patients residing in zip codes with lower-income or predominately racial/ethnic minority populations. The extent to which access barriers to telemedicine contribute to lower rates of in-person care deferral and thus increases in potential exposure to COVID-19 should be examined in future work.

### Limitations

This study is not without limitations. First, while we used claims from a large and diverse study population, it represents only a subset of individuals with private insurance and does not include other important populations, such as patients with Medicare or Medicaid and those lacking insurance. By definition, our population receives insurance through an employer. Thus, our findings of disparities are among those who are employed and do not represent the likely full extent of disparate responses to the COVID-19 pandemic. Second, we did not examine whether care that has been deferred in the earliest days of the COVID-19 pandemic will be deferred until the future or avoided completely. Future work should continue to monitor the utilization trends and disparities in those trends. Finally, we did not examine the association of social distancing policies, such as shelter-in-place mandates, with reductions in health care use, but future work should test how patients and health professionals respond to these policies.^35,36,37,38^

The consequences of the observed reductions in care on patient health will critically depend on the persistence of care deferrals. If the reductions in care we observed are delayed by a few months and then return to previous levels, as some evidence suggests,^39^ then patient health impacts may be limited, and the short-run declines in medical spending may lead to reduced annual 2020 medical spending. However, extending care deferrals for many months, or even perhaps until a COVID-19 vaccine is developed could substantially harm patient health. We do not examine the health impacts of delayed care, but future work should test how delayed care due to COVID-19 and related policies has affected patient health outcomes. If delayed care worsens patient health, then the short-run savings we observe may be illusory if patients require more intensive and costly treatment in the coming months and years.

## Conclusions

The results of this cross-sectional study highlight the profound shock to the health care delivery system created by the COVID-19 pandemic. If the current trends continue, innovative approaches to ensure patients receive timely access to important care will be required. These approaches will require collaboration from multiple stakeholders, including patients, health professionals, insurers, employers, and regulators.

## Notes

Supplement. **eAppendix.** Description of Data Sample and Telemedicine Procedures and Description of Regression Model **eFigure.** Unadjusted Utilization of Preventive, Non-elective, Elective, and Pharmaceutical Services in January/February and March/April 2020 Compared to 2018 **eTable 1.** Comparison Between Castlight and ACS Populations **eTable 2.** Industry Distribution **eTable 3.** Overall Change in Procedure Volume **eTable 4.** Multivariable Regression Results: Change in Health Care Service Spending in March and April 2020 **eTable 5.** Multivariable Regression Results: Change in Office Visit and Telemedicine Use in March and April 2020 **eTable 6.** Regression-Adjusted Changes in Medical Utilization by Zip Code Income and Race
