# Supplementary material for: Changes in Health Services Use Among Commercially Insured US Populations During the COVID-19 Pandemic
Source: JAMA Netw Open. 2020 Nov 5;3(11):e2024984. doi: 10.1001/jamanetworkopen.2020.24984 (PMC7645698; doi:10.1001/jamanetworkopen.2020.24984)
Supplement: Supplement. — eAppendix. Description of Data Sample and Telemedicine Procedures and Description of Regression Model eFigure. Unadjusted Utilization of Preventive, Non-elective, Elective, and Pharmaceutical Services in January/February and March/April 2020 Compared to 2018 eTable 1. Comparison Between Castlight and ACS Populations eTable 2. Industry Distribution eTable 3. Overall Change in Procedure Volume eTable 4. Multivariable Regression Results: Change in Health Care Service Spending in March and April 2020 eTable 5. Multivariable Regression Results: Change in Office Visit and Telemedicine Use in March and April 2020 eTable 6. Regression-Adjusted Changes in Medical Utilization by Zip Code Income and Race [file jamanetwopen-e2024984-s001.pdf]

Supplemental Online Content

Whaley CM, Pera MF, Cantor J, et al. Changes in health services use among commercially insured US populations during the COVID-19 pandemic. *JAMA Netw Open*. 2020;3(11):e2024984. doi:10.1001/jamanetworkopen.2020.24984

eAppendix. Description of Data Sample and Telemedicine Procedures and Description of Regression Model
eFigure. Unadjusted Utilization of Preventive, Non-elective, Elective, and Pharmaceutical Services in January/February and March/April 2020 Compared to 2018
eTable 1. Comparison Between Castlight and ACS Populations
eTable 2. Industry Distribution
eTable 3. Overall Change in Procedure Volume
eTable 4. Multivariable Regression Results: Change in Health Care Service Spending in March and April 2020
eTable 5. Multivariable Regression Results: Change in Office Visit and Telemedicine Use in March and April 2020
eTable 6. Regression-Adjusted Changes in Medical Utilization by Zip Code Income and Race

This supplemental material has been provided by the authors to give readers additional information about their work.

© 2020 Whaley CM et al. *JAMA Network Open.*

eAppendix 1. Description of Data Sample

This study used medical and pharmacy claims data from employers who purchased access to the Castlight Health platform, which provides price transparency, wellness and other health benefits tools. This analysis did not include the digital tools provided by Castlight Health, but instead used the claims data that participating employers provide to Castlight as a way to implement the digital tools. For each of approximately 200 self-insured employers that provide access to this tool, the claims data covers all in-network procedures that are reimbursed through insurance. The claims data includes reimbursement amounts, procedure codes, and patient diagnoses. The data also includes demographic (e.g., geographic location, age, gender) and employer information (e.g., industry). We did not have access to individual-level data, but instead data aggregated to the year-month-state-gender-age group category level.

One potential concern is that the population included in this sample may not be representative of the broader U.S. population. To assess differences between our study population and the broader commercially insured population, we used data from the American Community Survey (ACS). 2018 is the most recent year available in the ACS, and so we limited our comparison to 2018. We limited the ACS sample to individuals who receive insurance through an employer or union and are under the age of 65. We applied the nationally representative population weights in the ACS data. As shown in Table A1, the Castlight population is similar in gender, age, and geographic distribution to the ACS population.

Definition of Telemedicine procedures

Telemedicine procedures were identified as claims with procedure codes in the following set: ('99441','99442','99443','99444','99421','99422','99423','98970','98971','98972','G2061','G2062','G2063'), claims with a procedure code modifier in ('95','GT','GQ'), or a place of service code equal to 2.

Description of Regression Model

To examine the association between health care utilization and the first month of the COVID-19's declaration of a national emergency in the U.S., we estimate a regression model that quantifies the change in health care utilization in March 2020, relative to previous periods. We defined healthcare utilization by measuring the number of persons per 10,000 persons who received each of the 145 services grouped by the IBM Watson Health procedure categories. We defined utilization rates ($rate_{ikst}$) for procedure k received by patients (age and gender-level) i who live in state s during time period (year and month) t. Ages are categorized as 0-2, 3-18, 19-26, 27-45, and 46-64. For colonoscopies, we restricted the denominator population to persons ages 46 to 64; for mammograms, women ages 46 to 64; and for infant vaccines, children ages 0 to 2.

With these utilization measures we estimated a regression model of the form

© 2020 Whaley CM et al. *JAMA Network Open.*

$$rate_{ikst} = \alpha + \delta March2020_t + \beta_1 age_i + \beta_2 gender_i + \beta_3 state_s + \beta_4 year_t + \beta_5 month_t + \varepsilon_{ikst}$$

In this model, the $March2020_t$ term is an indicator for the March 2020 time period. We included fixed effects controls for the age categories, gender, state, year, and month. We estimated this model separately for the 10 procedures of interest. For the model where we pooled across procedures, we included a fixed effect for procedure ($\beta_6 procedure_k$). We estimated this model using linear regressions with Stata version 16.

For the models that assess differences by the five-digit zip code level income and race, we first linked the utilization/ measures to zip code. We then used data from the 2018 American Community Survey (ACS) on zip code level household income and race. For income, we defined zip code mean income relative the federal poverty line (FPL) for a family of four ($26,200). We categorized mean zip code income as below 200% of FPL ($52,400), between 200% and 400% ($104,800) and above 400% of FPL. For race, we categorized zip codes as those with 80% or more non-white residents, 79% to 21% non-white residents, and 80% or more white residents (and 20% or fewer non-white residents).

To measure the differences in changes in health care utilization after the national emergency declaration based on zip code income, we estimated a regression model of the form

$$rate_{ikst} = \alpha + \delta_1 March2020_t + \delta_2 income_1 + \delta_3 income_2 + \delta_4 March2020_t \times income_1 + \delta_5 March2020_t \times income_2 + \beta_1 age_i + \beta_2 gender_i + \beta_3 state_s + \beta_4 year_t + \beta_5 month_t + \varepsilon_{ikst}$$

In this model, $income_1$ represents zip codes with mean household income below 200% of FPL and $income_2$ represents zip codes with household income between 200% and 400% of FPL. The omitted category is zip codes with mean household income 400% or more of FPL. The regression model includes indicator controls for the income level, plus interaction terms between each income level and the March 2020 indicator.

To measure the differences in changes in health care utilization after the national emergency declaration based on the percent of the zip code that is non-white, we estimated a regression model of the form

$$rate_{ikst} = \alpha + \delta_1 March2020_t + \delta_2 race_1 + \delta_3 race_2 + \delta_4 March2020_t \times race_1 + \delta_5 March2020_t \times race_2 + \beta_1 age_i + \beta_2 gender_i + \beta_3 state_s + \beta_4 year_t + \beta_5 month_t + \varepsilon_{ikst}$$

In this model, $race_1$ represents zip codes 80% or more non-white residents and $race_2$ represents zip codes with between 79% and 21% non-white residents. The omitted category is zip codes with 80% or more white residents. The regression model includes indicator controls for the race category, plus interaction terms between each race category and the March 2020 indicator.

© 2020 Whaley CM et al. *JAMA Network Open.*

eFigure 1: Unadjusted Utilization of Preventive, Non-elective, Elective, and Pharmaceutical Services in January/February and March/April 2020 Compared to 2018

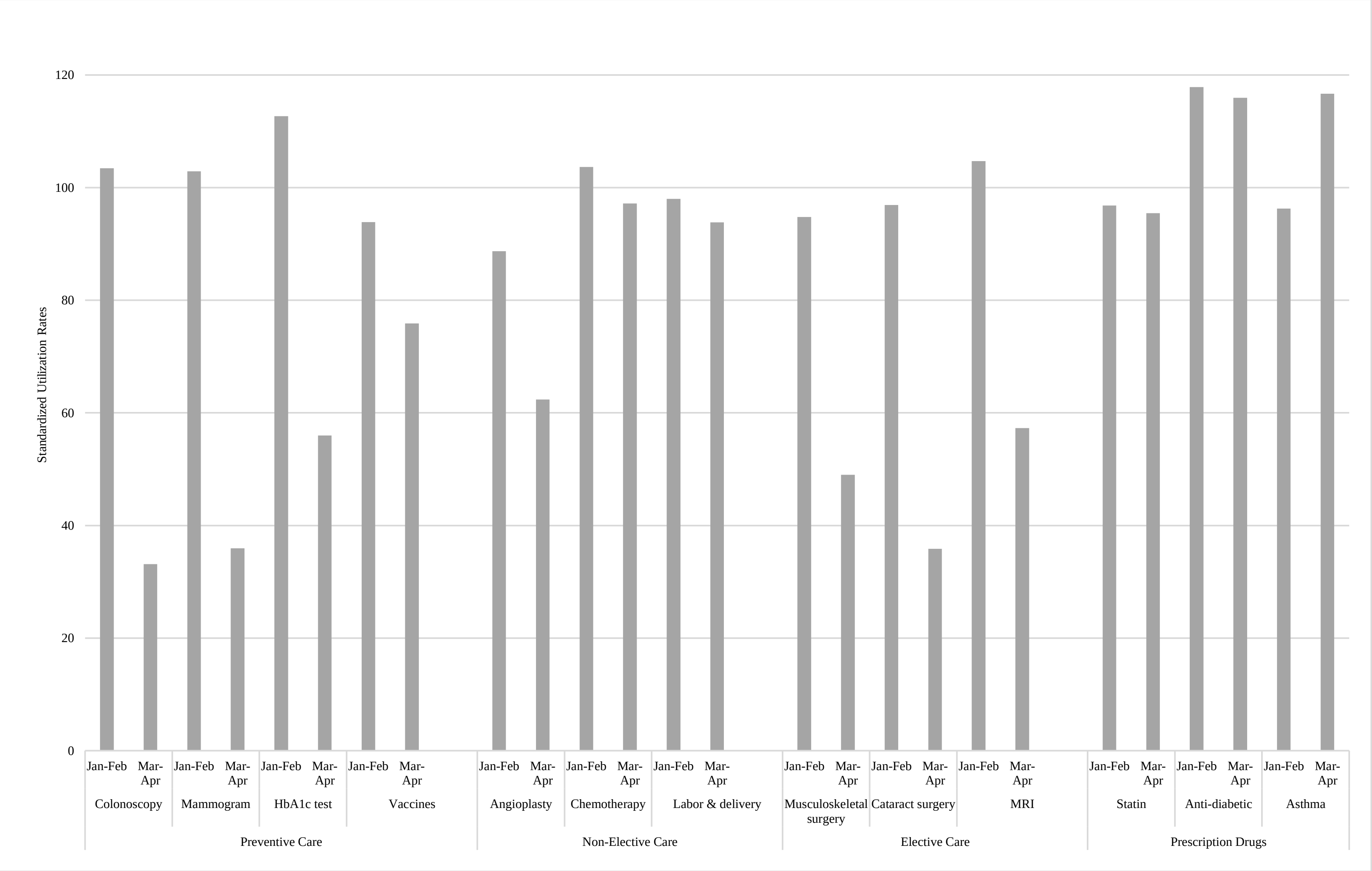

Note: Colonoscopy population limited to ages 46-64, mammogram population limited to women ages 46-64, vaccine population limited to children ages 0-2, and labor and delivery population limited to women ages 19-45.

© 2020 Whaley CM et al. *JAMA Network Open.*

eTable 1: Comparison Between Castlight and ACS Populations

Study year	Castlight (2018)	ACS (2018)
Number of enrolled persons	5,608,888	162,136,077
Gender, percent female (%)	50.0%	50.0%
Average age	34.3	33.5
Census region		
South	40.6%	35.70%
Midwest	23.0%	22.80%
Northeast	10.4%	18.20%
West	26.0%	23.40%

© 2020 Whaley CM et al. *JAMA Network Open.*

We also examined the industry distribution of the Castlight population, across 30 industries. As presented in eTable 2, most enrollees receive insurance through an employer in the telecommunications industry.

eTable 2: Industry Distribution

Industry	Share of enrollees
Telecommunications	13.31%
Aerospace & Defense	12.24%
Retail	10.65%
Government	8.78%
Manufacturing	7.05%
Financial Services	6.65%
Automotive	5.32%
Food & Beverage	4.79%
Insurance	4.79%
Hospitals & Healthcare	4.52%
Other	3.19%
Grocery	2.79%
Oil, Energy, & Utilities	2.79%
Education	2.26%
Semiconductors	1.46%
Pharmaceuticals & Biotech	1.32%
Electronics	1.26%
Transportation	1.21%
Professional Services	1.11%
Entertainment & Hospitality	0.80%
Medical Devices	0.75%
Construction	0.71%
Software & Technology	0.65%
Non-Profit	0.47%
Engineering	0.37%
Chemicals	0.28%
Security	0.28%
Real Estate	0.08%
Legal Services	0.05%
Media	0.04%

© 2020 Whaley CM et al. *JAMA Network Open.*

eTable 3: Overall Change in Procedure Volume

	(1)
	All Procedures
Change in incidence per 10,000 persons during March 2020	-14.01***
	(-14.81 to -13.20)
Change in incidence per 10,000 persons during April 2020	-32.16***
	(-32.96 to -31.36)
Observations	2,898,405
R-squared	0.757
March mean	61.8
April mean	62.4
Relative change between March 2019 and March 2020	-22.7%
Relative change between April 2019 and April 2020	-51.5%

This table shows regression-adjusted differences in utilization of all 145 medical procedures grouped by the IBM Watson Health procedure categories in March and April 2020, relative to the 2018 to 2020 time period. The dependent variable in each column is the monthly number of persons per 10,000 eligible persons with the respective procedure. Regression models include fixed effect controls for year and month, state, patient gender, and age category (categorized as 0-2, 3-18, 19-26, 27-45, 46-64).

95% Confidence intervals in parentheses. ** p<0.01, * p<0.05

© 2020 Whaley CM et al. *JAMA Network Open.*

eTable 4: Multivariable Regression Results: Change in Health Care Service Spending in March and April 2020

	(1)	(2)	(3)	(4)	(5)	(6)	(7)	(8)	(9)	(10)	(11)	(12)	(13)
				Preventive Care		Non-Elective Care		Elective Care					
	All Procedures	Colonoscopy	Mammogram	HbA1C test	Vaccines	Angioplasty	Chemotherapy	Child delivery	Musculoskeletal surgery	Cataract surgery	MRI	Office visits	Telemedicine
Change in mean per-member monthly spending during March 2020 ($)	-0.494***	-5.705***	-5.709***	-9.242***	-0.107***	-0.0695	-0.161	-0.0637	-1.713***	-0.332***	-2.860***	-13.03***	2.875***
	(-0.586 to -0.401)	(-6.462 to -4.949)	(-6.308 to -5.110)	(-11.33 to -7.159)	(0.00515)	(-1.139 to 1.000)	(-0.641 to 0.319)	(-0.148 to 0.0208)	(-1.961 to -1.465)	(-0.453 to -0.211)	(-3.546 to -2.175)	(-14.24 to -11.81)	(2.628 to 3.122)
Change in mean per-member monthly spending during April 2020 ($)	-1.063***	-12.66***	-11.78***	-10.32***	-0.192***	-0.484	-0.196	-0.109**	-3.741***	-0.927***	-6.760***	-27.64***	10.72***
	(-1.153 to -0.972)	(-13.55 to -11.77)	(-12.53 to -11.03)	(-12.48 to -8.166)	(0.00513)	(-1.470 to 0.503)	(-0.674 to 0.282)	(-0.192 to -0.0255)	(-4.049 to -3.434)	(-1.047 to -0.807)	(-7.629 to -5.891)	(-29.17 to -26.12)	(10.02 to 11.42)
Observations	2,898,405	2,856	5,712	2,853	19,989	2,856	19,989	19,989	19,989	19,989	19,989	19,989	19,989
R-squared	0.598	0.800	0.737	0.897	0.817	0.648	0.667	0.132	0.770	0.708	0.654	0.842	0.782
March mean	$2.30	$12.34	$11.27	$55.26	$0.27	$10.82	$5.49	$0.30	$5.49	$0.83	$8.93	$39.98	$0.11
April mean	$2.34	$13.16	$12.12	$55.01	$0.26	$11.01	$5.88	$0.24	$5.70	$0.96	$9.29	$40.20	$0.11
Relative change between March 2019 and March 2020	-21.5%	-46.2%	-50.7%	-16.7%	-40.3%	-0.6%	-2.9%	-21.3%	-31.2%	-40.2%	-32.0%	-32.6%	2508.6%
Relative change between April 2019 and April 2020	-45.5%	-96.2%	-97.2%	-18.8%	-73.2%	-4.4%	-3.3%	-44.7%	-65.7%	-96.3%	-72.8%	-68.8%	9539.8%

This table shows regression-adjusted differences in per-member, per-month spending on all procedures, preventive, non-elective care, elective care, in-person office visits, and telemedicine in March and April 2020, relative to the 2018 to 2020 time period. Regression models include fixed effect controls for year and month, state, patient gender, and age category (categorized as 0-2, 3-18, 19-26, 27-45, 46-64). Column 1 (All procedures) includes fixed effects for procedure type.

Note: Colonoscopy population limited to ages 46-64, mammogram population limited to women ages 46-64, vaccine population limited to children ages 0-2, and labor and delivery population limited to women ages 19-45. 95% Confidence intervals in parentheses. ** p<0.01, * p<0.05

© 2020 Whaley CM et al. *JAMA Network Open.*

eTable 5: Multivariable Regression Results: Change in Office Visit and Telemedicine Use in March and April 2020

	(1)	(2)
	Office visits	Telemedicine
Change in incidence per 10,000 persons during March 2020	-581.1***	227.9***
	(-612.9 to -549.3)	(221.7 to 234.1)
Change in incidence per 10,000 persons during April 2020	-1,465***	641.6***
	(-1,496 to -1,433)	(635.5 to 647.8)
Observations	19,989	19,989
R-squared	0.873	0.845
March mean	2237.2	17.4
April mean	2236.9	15.6
Relative change between March 2019 and March 2020	-26.0%	1306.2%
Relative change between April 2019 and April 2020	-65.5%	4119.7%

This table shows regression-adjusted differences in use of office visits and telemedicine in March and April 2020, relative to the 2018 to 2020 time period. The dependent variable in each column is the monthly number of persons per 10,000 eligible persons with the respective procedure. Regression models include fixed effect controls for year and month, state, patient gender, and age category (categorized as 0-2, 3-18, 19-26, 27-45, 46-64).
95% Confidence intervals in parentheses. ** p<0.01, * p<0.05

© 2020 Whaley CM et al. *JAMA Network Open.*

eTable 6: **Regression-Adjusted Changes in Medical Utilization by Zip Code Income and Race**

	(1)	(2)	(3)	(4)	(5)	(6)
	Colonoscopy	Mammogram	HbA1C	Vaccines	Office visits	Telemedicine
Panel A: Income Differences						
Pre Covid difference in utilization for persons in zip codes with income below 200% of FPL, relative to persons in zip codes with income above 400% FPL	-15.01***	-80.21***	-392.0***	9.293***	-302.1***	-2.311*
	(-16.05 to -13.98)	(-85.27 to -75.14)	(-412.0 to -371.9)	(7.864 to 10.72)	(-312.8 to -291.5)	(-4.628 to 0.00636)
Pre Covid difference in utilization for persons in zip codes with income between 201-400% of FPL, relative to persons in zip codes with income above 400% FPL	-8.443***	-46.75***	-104.3***	0.348	-146.5***	-0.655
	(-9.319 to -7.568)	(-51.07 to -42.43)	(-120.2 to -88.41)	(-0.854 to 1.549)	(-155.4 to -137.5)	(-2.603 to 1.293)
Change in utilization in March/April 2020	-54.80***	-281.1***	-458.4***	-102.1***	-1,276***	519.9***
	(-57.70 to -51.90)	(-295.5 to -266.7)	(-511.7 to -405.1)	(-106.1 to -98.15)	(-1,306 to -1,246)	(513.4 to 526.4)
Change in utilization in March/April 2020 for persons in zip codes with income below 200% of FPL, relative to change in zip codes with income above 400% FPL	14.11***	56.06***	241.8***	21.56***	441.9***	-153.3***
	(10.90 to 17.31)	(40.30 to 71.82)	(179.3 to 304.2)	(17.12 to 25.99)	(408.8 to 475.1)	(-160.5 to -146.1)
Change in utilization in March/April 2020 for persons in zip codes with income between 201-400% of FPL, relative to change in zip codes with income above 400% FPL	7.916***	34.05***	121.1***	13.68***	236.0***	-77.55***
	(5.074 to 10.76)	(19.94 to 48.17)	(69.14 to 173.1)	(9.772 to 17.58)	(206.8 to 265.2)	(-83.88 to -71.22)
Panel B: Race Differences						
Pre Covid difference in utilization for persons in zip codes with 80%+ nonwhite residents, relative to persons in zip codes with 80%+ white residents	-9.113***	-39.35***	-414.8***	58.89***	-562.0***	-9.039***
	(-11.15 to -7.071)	(-48.66 to -30.04)	(-455.9 to -373.7)	(55.89 to 61.89)	(-583.4 to -540.6)	(-13.92 to -4.161)
Pre Covid difference in utilization for persons in zip codes with 79-21% nonwhite residents, relative to persons in zip codes with 80%+ white residents	-4.178***	-15.13***	-72.87***	19.59***	-191.3***	-2.369***
	(-4.825 to -3.530)	(-18.29 to -11.98)	(-84.88 to -60.86)	(18.64 to 20.55)	(-198.1 to -184.5)	(-3.923 to -0.815)
Change in utilization in March/April 2020	-48.38***	-249.2***	-348.9***	-81.65***	-1,056***	444.5***
	(-50.31 to -46.45)	(-258.7 to -239.7)	(-385.5 to -312.2)	(-84.54 to -78.76)	(-1,077 to -1,035)	(439.8 to 449.2)
Change in utilization in March/April 2020 for persons in zip codes with 80%+ nonwhite residents, relative to change in zip codes with 80%+ white residents	6.925**	5.505	73.11	-41.57***	199.5***	-71.56***
	(0.161 to 13.69)	(-25.55 to 36.56)	(-60.86 to 207.1)	(-51.47 to -31.68)	(128.9 to 270.1)	(-87.64 to -55.47)
Change in utilization in March/April 2020 for persons in zip codes with 79-21% nonwhite residents, relative to change in zip codes with 80%+ white residents	3.697***	6.449	26.24	-12.23***	54.24***	-15.08***
	(1.736 to 5.658)	(-3.177 to 16.08)	(-10.41 to 62.89)	(-15.12 to -9.333)	(33.62 to 74.87)	(-19.78 to -10.38)

This table shows regression-adjusted differences in use rates of preventive and primary care (office visit and telemedicine) in March/April 2020, relative to the 2018 to 2020 time period, and how these differences vary by zip code level income (Panel A) and race (Panel B). Zip code race and income defined using data from the American Community Survey. For income, we defined zip code mean income relative the federal poverty line for a family of four ($26,200). We categorized mean zip code income as below 200% of FPL ($52,400), between 200% and 400% ($104,800) and above 400% of FPL. For race, we categorized zip codes as those with 80% or more non-white residents, 79% to 21% non-white residents, and 80% or more white residents (and 20% or fewer non-white residents). The dependent variable in each column is the monthly number of persons per 10,000 eligible persons with the respective procedure. Regression models include fixed effect controls for year and month, state, patient gender, and age category (categorized as 0-2, 3-18, 19-26, 27-45, 46-64).
Note: Colonoscopy population limited to ages 46-64, mammogram population limited to women ages 46-64, and vaccine population limited to children ages 0-2.
95% Confidence intervals in parentheses. ** p<0.01, * p<0.05

© 2020 Whaley CM et al. *JAMA Network Open.*
